# Identification and Characterization of Epithelial Cell-Derived Dense Bodies Produced upon Cytomegalovirus Infection

**DOI:** 10.3390/vaccines10081308

**Published:** 2022-08-12

**Authors:** Estéfani García-Ríos, María Josefa Rodríguez, María Carmen Terrón, Daniel Luque, Pilar Pérez-Romero

**Affiliations:** 1 National Center for Microbiology, Instituto de Salud Carlos III, Majadahonda, 28220 Madrid, Spain; 2 Department of Science, Universidad Internacional de Valencia—VIU, Pintor Sorolla 21, 46002 Valencia, Spain; 3 Electron and Confocal Microscopy Unit, Instituto de Salud Carlos III, Unidades Centrales Científico-Técnicas, Majadahonda, 28220 Madrid, Spain

**Keywords:** cytomegalovirus, dense bodies, vaccine design, epithelial cells, TEM

## Abstract

Dense bodies (DB) are complex, noninfectious particles produced during CMVinfection containing envelope and tegument proteins that may be ideal candidates as vaccines. Although DB were previously described in fibroblasts, no evidence of DB formation has been shown after propagating CMV in epithelial cells. In the present study, both fibroblast MRC-5 and epithelial ARPE-19 cells were used to study DB production during CMV infection. We demonstrate the formation of epithelial cell-derived DB, mostly located as cytoplasmic inclusions in the perinuclear area of the infected cell. DB were gradient-purified, and the nature of the viral particles was confirmed using CMV-specific immunelabeling. Epithelial cell-derived DB had higher density and more homogeneous size (200–300 nm) compared to fibroblast-derived DB (100–600 nm).In agreement with previous results characterizing DB from CMV-infected fibroblasts, the pp65 tegument protein was predominant in the epithelial cell-derived DB. Our results also suggest that epithelial cells had more CMV capsids in the cytoplasm and had spherical bodies compatible with nucleus condensation (pyknosis) in cells undergoing apoptosis that were not detected in MRC-5 infected cells at the tested time post-infection. Our results demonstrate the formation of DB in CMV-infected ARPE-19 epithelial cells that may be suitable candidate to develop a multiprotein vaccine with antigenic properties similar to that of the virions while not including the viral genome.

## 1. Introduction

Cytomegalovirus (CMV) is a ubiquitous herpesvirus that establishes benign infection in immunocompetent hosts, producing no or mild symptoms. However, in individuals with an immature or dysfunctional immune system, such as neonates and immunocompromised patients, CMV is a major cause of morbidity and mortality, such as HIV-infected patients and transplant recipients, in which the infection can cause severe symptoms [1,2,3,4,5].

The mature CMV virions are 200nm diameter particles with 235 Kb double-stranded linear DNA genome inside a 130nm icosahedral capsid and surrounded by a lipid envelope, with a thick tegument protein layer between the nucleocapsid and the envelope [6]. CMV-infected cells produce in abundance complex enveloped sub-viral particles called dense bodies (DB) that include the envelope and the tegument, the obtained quantities of these CMV particles are dependent on the viral strain used and the multiplicity of infection [7,8,9].

Fibroblasts are the most extended cell-culture laboratory model used for CMV propagation, due to a high replication rate producing higher viral titers compared to other cell types. Most studies characterizing DB are performed in fibroblasts infected with different CMV strains such as AD169 CMV strain defective in the expression of the pentameric complex (PC), AD169 laboratory strain in which the PC was repaired [10,11,12,13,14], endotheliotropic strains [8,15] or Towne strain [16], among others. However, epithelial and endothelial cells are the main targets of CMV during natural infection, and it is likely that CMV propagation in epithelial cells might affect differently the final composition of the virions, i.e., in glycosylation sites or protein level and protein composition, and therefore in their antigenicity and the induction of the immune response [17]. In fact, it has been described that minimal changes in the CMV virion protein composition may have a significant impact on antigenicity and thus in the capacity of eliciting a protective immune response [12,13]. In this sense, many of the fibroblast-derived DB particles might lack the PC UL128-131 proteins that are not necessary during cell entry in this cell type, which may not be ideal for eliciting a complete neutralizing antibody response able to block infection in epithelial cells.

Although the development of a vaccine against CMV has been identified as a top-priority by the National Vaccine Program Office and the Institute of Medicine of the United States [18,19], and beside the efforts made in recent decades, no vaccine has yet been commercialized [20,21]. In the context of the SARS-CoV-2 pandemic, the development of an effective vaccine against CMV has been highlighted due to the higher pathogen susceptibility of immune-compromised patients, such as transplant recipients [22,23,24].

The envelope glycoproteins gB (UL55), gH (UL75) and gN (UL73) and the PC proteins involved in the recognition of the cellular receptors and entry into cells have been proposed as the main targets for neutralizing antibodies [25,26,27,28]. The PC is involved in the recognition of the cellular receptor during virus entry in epithelial and endothelial cells and has been identified as the main target of neutralizing antibodies [17,25,29,30]. Thus, some of the vaccine candidates were designed to produce a complex immune response, comparable to that of natural infection using replication-defective or attenuated viruses [31] or noninfectious subviral particles [12,13,14]. However, these approaches had some limitations, such as safety issues because they contain the viral genome, or producing partial immune response lacking important neutralizing antibodies, respectively.

A DB-based vaccine may offer several benefits: they are non-infectious particles since they do not contain a viral genome, which is an additional safety advantage for a vaccine. DB have a protein composition similar to that of complete virus particles, with an important number (around 20) of common proteins, including envelope glycoproteins and tegument proteins [8,9,11,32,33]. DB are formed in the host cell cytoplasm with envelopment and release steps similar to virions [34] with viral proteins integrated in their functional conformation into the envelope, which may likely produce high levels of neutralizing antibodies and antibodies with other effector functions against CMV infection [11,13,14,15,35]. In fact, the humoral response elicited by DB is comparable to that induced by viral infection [13,35], except for the neutralizing antibody response. Due to the lack of evidence describing the formation of DB in epithelial cells and on the previously stated, using DB produced in epithelial cells that will include the PC proteins may be a better strategy to develop a multiprotein vaccine against CMV with antigenic properties similar to that of the epitheliotropic virions.

Here, we report the existence, identification and initial characterization of DB in ARPE-19 epithelial cells by means of transmission electron microscopy (TEM) and biochemical techniques.

## 2. Material and Methods

### 2.1. Cells, Viruses and Viral Infections

MRC-5 fibroblast and ARPE-19 epithelial cells were obtained from the American Type Culture Collection (ATCC; Manassas, VA, USA) and cultured as recommended. The CMV strain used in this study was BADrUL131-Y4, which is derived from the AD169 strain in which the UL131 sequence was repaired [36], kindly provided by Dr. Shenk (Princeton University). MRC-5 and ARPE-19 cells were cultured in flasks with DMEM supplemented with 10% fetal bovine serum (FBS), 20 mM glutamine (Lonza, Basilea, Suiza) and 10 units of Penicillin and 10 μg of Streptomycin (Lonza, Basilea, Suiza). Cells were infected at a multiplicity of infection (MOI) of 0.01 PFU/cell when they reached 85–90% confluency.

### 2.2. Dense Bodies’Purifications and Protein Analysis

Culture supernatants were collected from three 175 cm flasks of CMV-infected MRC-5 and ARPE-19 cells, at 10 days after infection (at 0.01 MOI), and processed for recovery of noninfectious enveloped particles (NIEPs), virions, and DB using glycerol-tartrate gradients as previously reported [32,33]. When the gradient was illuminated from above, the upper band corresponding to virions and the lower band corresponding to DB presented distinct light scattering. The gradients were 0.9 mL-fractionated, and the protein composition of each fraction was analyzed by 12% SDS polyacrylamide gel electrophoresis and visualized by Coomassie brilliant blue staining (BioRad, Hercules, CA, USA). All gradient fractions, containing DB and virions, were diluted in TNE buffer (50 mM Tris [pH 7.4], 100 mM NaCl, and 10 mM EDTA), pelleted by centrifugation at 50,000× *g* for 2h at 14 °C in a Beckman SW60 rotor and resuspended in 100 μL of TNE buffer. Purity was confirmed by electron microscopy of negatively stained HCMV preparations. Protein pellets were lysed, suspended in Laemmli buffer and separated on a 12% SDS gel. After transferring to nitrocellulose membranes, the blots were probed with an anti-cytomegalovirus polyclonal antibody (diluted 1:500; CA150-1, Virusys, Randallstown, MD, USA) followed by a horseradish peroxidase (HRP)-labeled anti-chicken IgG (diluted 1:2000; clone 8B1.2, Sigma, Saint Louis, MO, USA) and detected using chemiluminescence (SuperSignal West Pico Detection Kit, Thermo Scientific, Waltham, MA, USA).

The size distribution of DB was estimated by direct measurements with the measurement tool of a FEI Tecnai 12 electron microscope equipped with a LaB6 filament and operated at 120 kV.

### 2.3. Analysis of Virus Replication

Infection experiments in MRC-5 and ARPE-19 cells were performed at a 0.01 MOI, as indicated in the previous section. Viral replication kinetics were analyzed by quantitative real-time PCR (qPCR), as described previously [37]. Standardized results were expressed as international units per milliliter.

### 2.4. Ultrastructural Analysis of CMV-Infected Cells

Monolayers of MRC-5 and ARPE-19 cells were grown on coverslips and infected at a 0.01 MOI. At 10 days post-infection, the cells were fixed in situ for 1 h at room temperature (RT) with 2% paraformaldehyde (Ultra-Pure EM Grade, Polysciences Inc., Warrington, PA, USA) and 2.5% glutaraldehyde (EM Grade, TAAB Laboratories Equipment Ltd., Berks, UK) in PBS. Fixed monolayers were washed once with PBS and once with distilled water. Cell post-fixation was as follows: 45 min at RT with 1% osmium tetroxide (TAAB Laboratories Equipment Ltd. Berks, UK) in PBS, washed with distilled water, 45 min incubation at RT with 1% aqueous uranyl acetate (Electron Microscopy Sciences, Hatfield, PA, USA), and after dehydration with increasing concentrations of ethanol absolute anhydrous (VWR Chemicals BDH, Suwanee, GA, USA), cells were embedded in epoxy resin EML-812 (TAAB Laboratories Equipment Ltd. Berks, UK; 2 days, RT). Resin-containing gelatin capsules (TAAB Laboratories Equipment Ltd. Berks, UK) were placed on the coverslips and polymerized (2 days, 60 °C). Resin blocks were detached from coverslips by successive immersion in liquid nitrogen and hot water. Ultrathin 70 nm-thick sections, parallel to the monolayer, were obtained with a Leica EM UC6 ultramicrotome (Leica Microsystems GmbH, Wetzlar, Germany), transferred to Formvar-coated EM GS2x1-N3 nickel buttonhole grids and stained with 5% aqueous uranyl acetate (10 min, RT) and lead citrate (3 min, RT). Sections were visualized on a FEI Tecnai 12 electron microscope equipped with a LaB6 filament and operated at 120 kV. Images were recorded with a FEI Ceta digital camera at various magnifications.

### 2.5. Negative Staining

Samples were applied (5 min, RT) to glow-discharged carbon-coated grids and negatively stained with 2% aqueous phosphotungstic acid (PTA), or 1% sodium silicotungstate (SST) for 1 min at RT. For immunoelectron microscopy assays, after sample adsorption, grids were washed with TNE buffer; blocked in 1% BSA, 5% NG, PBS (10 min, RT); incubated with chicken anti-CMV antibody (clone 8B1.2, Sigma, Saint Louis, MO, USA) in blocking buffer (45 min, RT); washed 3 times with 0.1% BSA, 0.5% NGS, PBS (5 min, RT); incubated with goat anti-chicken antibody conjugated with 15nm colloidal gold (Electron Microscopy Sciences, Hatfield, PA, USA); diluted in washing buffer for 60 min at RT; washed 3 times with washing buffer (5 min, RT); 3 times with PBS (2 min, RT); and negatively stained for 1 min in 2% PTA or 1% SST. Samples were analyzed on a FEI Tecnai 12 electron microscope equipped with a LaB6 filament and operated at 120 kV. Images were recorded with a FEI Ceta digital camera at various magnifications.

### 2.6. Particle Size and Abundance Analysis

Fiji distribution of ImageJ program [38] was used both to measure the size of the DB purified from the fractions 9–11 of the gradients and to quantify the number of virus and DB particles per area unit in the cell cytoplasm. The diameter of 151 and 136 DB particles were measured in MRC-5 and ARPE-19 cells, respectively. To evaluate statistical significance between sizes, two-tailed t-Student test was applied with a *p*-value of 0.01.

## 3. Results

### 3.1. Ultrastructural Analysis of CMV-Infected MRC-5 and ARPE-19 Cells

**Figure 1:**
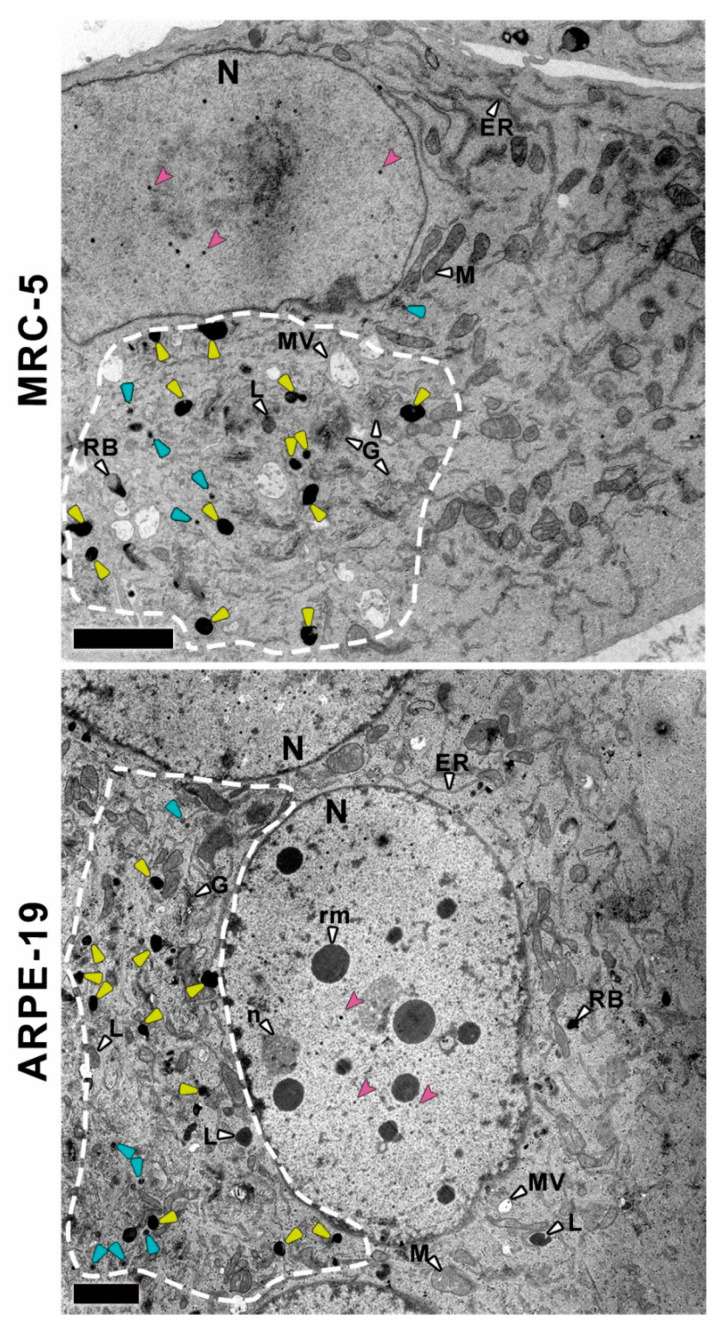
Panoramic view of CMV-infected MRC-5 and ARPE-19 in all legend cells.A cytoplasmic inclusion (CI; dashed lines) is located in the left half of the MRC-5 image, under the nuclear area. Proliferation of fragmented cisterna along with electron DB (yellow arrowheads) and viral capsids (blue arrowheads) can be observed, which are not visible in the half right. In the left side of the ARPE-19 cell image, a massive change in the cytoplasm corresponding to the CI (dashed lines) can be observed. CI consists of viral capsids (blue arrowheads), hundreds of membrane cistern (putative Golgi elements), apparently fragmented in shorter elements, and putative DB (yellow arrowheads), not appreciable in the right side. Nuclei from both MRC-5 and ARPE-19 cells contain CMV nucleocapsids (pink arrowheads). However, the ARPE-19-infected nucleus exhibits a cluster of electron dense round masses, not found in the nuclei from MRC-5-infected cells.Cellular organelles and structures are labeled with letters and pointed out with white arrowheads: N––nucleus, n––nucleolus, rm––electron dense round masses inside the nucleus, DB––dense bodies, L––liposomes, LB––lamellar bodies, RB––residual bodies, MV––multivesicular bodies, M––mitochondria, ER––endoplasmic reticulum, G––Golgi apparatus. Bars: 2 microns.

**Figure 2:**
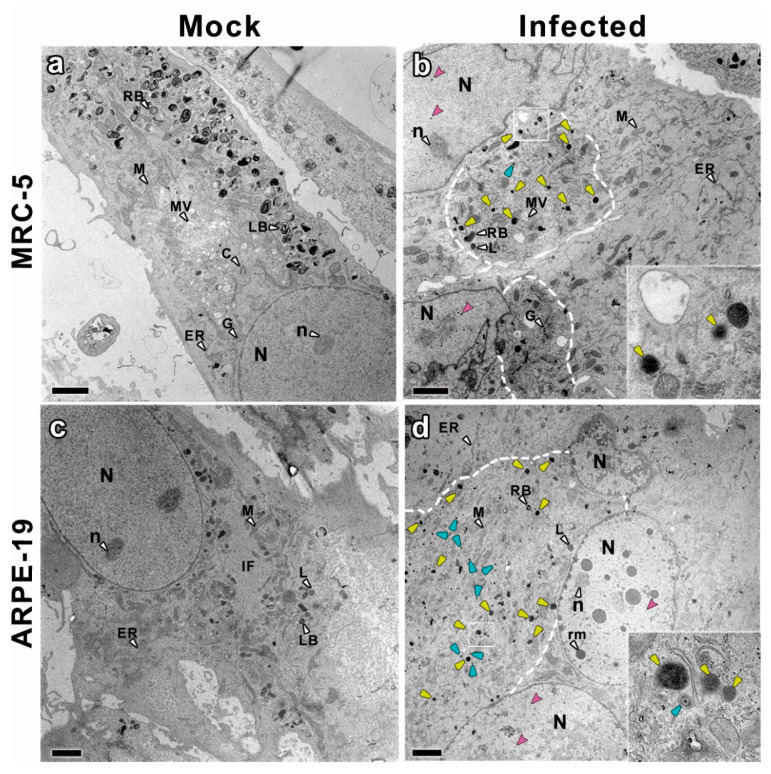
Electron microscopy images of epoxi-embedded sections of MRC-5 and ARPE-19 cells, mock and infectedsamples. (**a**) MRC-5 mock infected cells; (**b**) MRC-5 infected cells; (**c**) ARPE-19 mock-infected cells; (**d**) ARPE-19 infected cells. CI (dashed lines) alterations are observed in both infected cell types: more electron dense areas where there is an accumulation of components of the cellular secretory apparatus and structures compatible with DB (yellow arrowheads), along with viral particles (blue arrowheads). In the inset images, higher magnification of specific areas, labeled with squares, show structures compatible with DB, along with Golgi-like elements and viral particles. ARPE-19-infected cells show the formation of syncytia, multinucleated cells resulted from multiple cell fusions during the infection. Some of these nuclei exhibit multiple very electron dense round masses, associated at their periphery with viral capsids (pink arrowheads), and areas of electron opacity in contact with the nuclear membrane. Other cellular organelles and cellular structures are indicated with letters and white arrowheads: N––nucleus, n––nucleolus, rm––electron dense round masses inside the nucleus, L––liposomes, LB––lamellar bodies, RB––residual bodies, MV––multivesicular bodies, M––mitochondria, ER––endoplasmic reticulum, G––Golgi apparatus, IF––intermediate filaments. Bars: 2 microns.

In order to identify whether or not epithelial cells are able to produce and release DB upon CMV infection, CMV-infected MRC-5 and ARPE-19 cells were chemicallyfixed and their ultrastructure analyzed by TEM at 10 days post-infection. Fibroblast MRC-5 infected cells were chosen as controls, since DB were previously characterized in this cell line [10,11,12,13,14]. Infected cells showed clear alterations compared to mock infected cells (Figure 1 and Figure 2) including the assembly of nucleocapsids in the nucleus and the characteristic cytoplasmic inclusion (CI) of CMV [39]. It is noteworthy that ARPE-19 epithelial cells fused to produce large polykaryocytes, usually containing more than four nuclei that surround the CI (Figure 1 and Figure 2). This was not observed in MRC-5 infected cells at 10 days post-infection.

**Figure 3:**
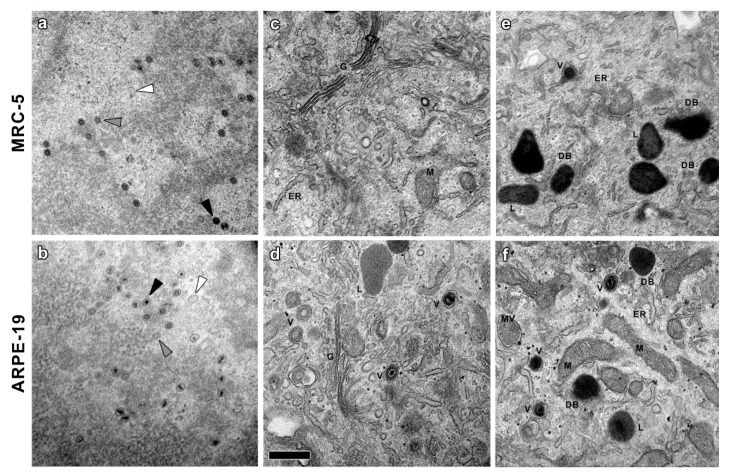
High magnification details of CMV-infected MRC-5 and ARPE-19 cells. (**a**,**b**) CMV nucleocapsids emerging from electron dense material (presumably nuclear inclusions) in the nucleoplasm. Empty A-capsids, scaffold-loaded B-capsids and DNA-loaded C-capsids are indicated with white, grey and black arrowheads, respectively. (**c**,**d**) Cytoplasmic areas with accumulation and proliferation of putative Golgi elements and capsids with tegument. (**e**,**f**) Electron-dense material similar to previously described DB, always located in cytoplasm areas rich in viral capsids and Golgi-like elements. Cellular organelles and structures are indicated: V––extranuclear viral capsids, DB––dense bodies, L––liposomes, MV––multivesicular bodies, M––mitochondria, ER––endoplasmic reticulum, G––Golgi apparatus. Bars: 500 nm.

Many nucleocapsids were observed in the nucleoplasm of both MRC-5 and ARPE-19 infected cells (Figure 1), including empty A-capsids, scaffold containing B-capsids and viral DNA containing C-capsids [16,34]. As shown in the TEM images, A-capsids were empty, the scaffold of B-capsids appeared as a ring and the DNA-filled C-capsids had an electrodense core (Figure 3a,b). These viral particles emerged from typical nuclear inclusions consisting of irregular strands of electron dense material, which were in close proximity to most of the developing capsids. At 10 days post-infection, some of the nuclei in the polykaryocytes showed a less electron-dense nucleoplasm with very electron dense spherical bodies associated at their periphery with viral capsids exclusively observed in ARPE-19 cells (Figure 1). The spherical bodies are compatible with nucleus condensation (pyknosis) in cells undergoing apoptosis [40,41].

In order to identify whether or not this difference was due to differences in cell replication rate, an infection kinetic wasperformed at 1, 5 and 10 days post-infection. The CMV titers obtained in both cell types are shown in Figure S1, which did not correlate with a faster CMV-replication kineticin ARPE-19 cells. The infected nuclei showed evenly distributed areas of electron opacity in contact with the nuclear membrane that could correspond to the infolding of the inner nuclear membrane [42].

The analysis of the CI in the surrounding of the nuclei in MRC-5 and ARPE-19-infected cells showed that they mainly consist of Golgi-like elements (apparently fragmented cisterna), viral particles and a high number of round dense masses compatible with DB (Figure 1 and Figure 3). This enlarged putative Golgi region had a higher electron density than the rest of the cytoplasm (Figure 1 and Figure 2, dashed lines) and, notably, with no presence of putative DB in other cytoplasmic regions. These CI areas are compatible with the previously described viral assembly complex, an accumulation of membranes and organelles that support the final steps in virus maturation and release [43]. At 10 days post-infection, ARPE-19 cells showed higher number of cytoplasmic CMV capsids than MRC-5 cells, in both cases located at the CI, and frequently acquiring envelopes by budding through the Golgi-like membranes (Figure 3).

**Figure 4:**
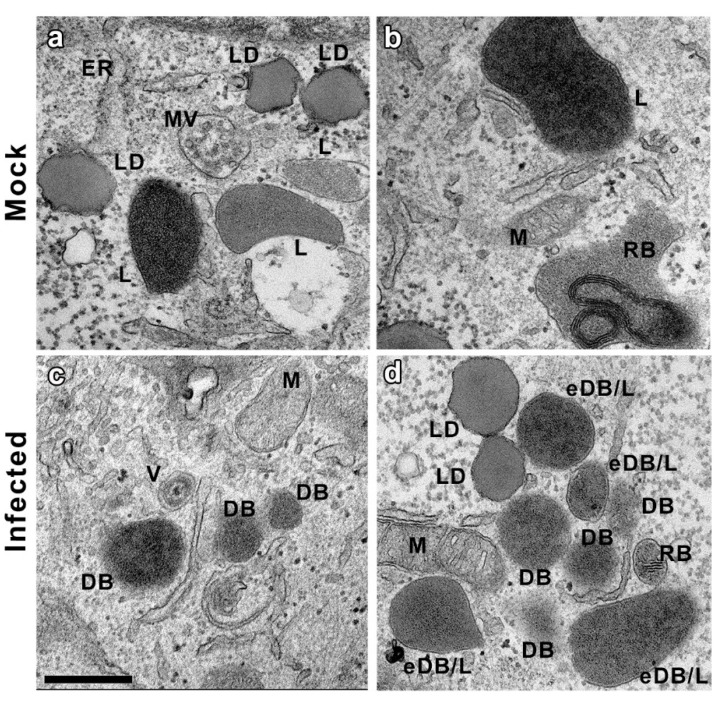
CMV DB identification. Comparison of cellular organelles and DB. (**a**,**b**) highlight structures found in mock infected ARPE-19 cells, most of them related with the cell endocytic processes. Lysosomes(L) are relatively electron-dense membrane-bound organelles, usually spherical and homogenously filled with tiny granules. Multivesicular bodies(MV) are round or oval cell organelles, usually containing an electron-lucent matrix, with small vesicles (diameter between 40 to 150 nm), also limited by adoublemembrane. Residual bodies (RB) or telolysosomes are surrounded by a membrane with very electron-dense granular content due to thefatty component of the lipofuscin(an aging pigment). Lipid droplets (LD) are composed of a neutral lipid core surrounded by a phospholipid monolayer. Note that all of them are membranous organelles. A mitochondrion (M) and endoplasmic reticulum (ER) were also detected. (**c**,**d**) show structures that can be found in infected ARPE-19 cells. In addition to all the previously described cellular organelles, DB structures, not present in mock samples, are identified. DB are similar in electron density to most of the lysosomes, but in many cases, with absence of membrane, and always surrounded by viral particles. Assemblies that were compatible with either enveloped DB or Lysosomes (eDB/L) were also detected. Bars: 500 nm.

The higher magnification of the CI (Figure 3e,f) revealed the presence of other organelles, mostly related with the cellular endocytic machinery similar to putative DB. However, the morphological characteristics of these structures, clearly visible at higher magnifications (beyond 15,000×), enabled us to distinguish the DB particles without complementary techniques (Figure 4). Lipid droplets (LD) had neutral lipids cores surrounded by a phospholipid monolayer. Multivesicular bodies (MV) were double-membrane organelles with an electron-lucent matrix and smaller vesicle inclusions. Residual bodies (RB) were very electron-dense granular content vesicles identified by the stacks of the fatty component of the lipofuscin. Lysosomes (L) and their different maturation derivatives were spherical vesicles with an electron density very similar to DB, hampering the unambiguous identification of these viral structures. However, while all of these are membranous organelles, DB can be either enveloped and non-enveloped [44]. It was therefore possible to unambiguously identify non-enveloped DB using exclusively morphological criteria, only detected in infected samples.

### 3.2. Purification and Characterization of Viral Particles

**Figure 5:**
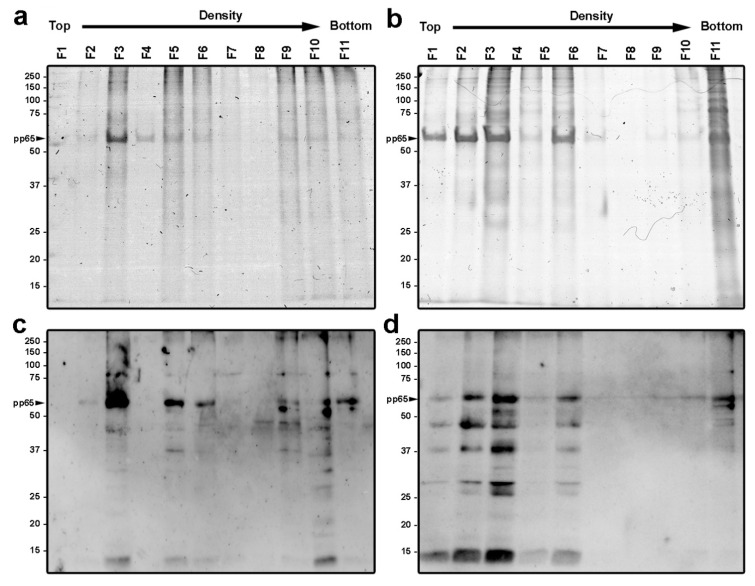
Protein composition of gradient fractions derived from MRC-5 and ARPE-19 infected cells. (**a**,**b**) Coomassie-staining SDS-PAGE analysis determining the protein distribution of each of the 11 fractions purified by centrifugation from supernatants of infected MRC-5 (**a**) and ARPE-19 (**b**) cells. Images were directly scanned using a Scanner Epson Perfection V500 photo. (**c**,**d**) Western blot analysis of the fractions using a polyclonal anti-CMV antibody. Lanes are indicated from 1 to 11, fraction 1 corresponding to the top (lower density) of the gradient tube and fraction 11 to the bottom (higher density). Analysis of the image was performed using a Chemidoc^TM^ MP Imaging System (Biorad, Hercules, CA, USA). Settings MRC-5: Exposure time (s) 122.124 (optimal autoexposure); Settings ARPE-19: Exposure time (s) 1.051 (rapid autoexposure). The expected migration of tegument pp65 protein is indicated with a black arrowhead.

As previously stated, published results has previously described enveloped DB produced from infected MRC-5 and other fibroblast cell lines [10,11,12,13,14,16,35]. However, this is the first report describing the detection of DB (enveloped or non-enveloped) in epithelial cells. To characterize the enveloped DB, glycerol-tartrate gradients were performed to separate the different viral particles isolated from the supernatant of the infected cells as described elsewhere [32,33]. Representative images of the obtained gradients are shown in Figure S2. Eleven fractions collected from MRC-5 and ARPE-19 derived gradients were characterized by Coomassie staining of SDS-PAGE gels. The protein profile of each of the eleven fractions demonstrated that fractions 3, 5 and 10–11 contained higher protein concentration in both cell lines (Figure 5a,b). Fractions were analyzed by Western blot using ananti-CMV polyclonal antibody (Figure 5c,d). Protein bands were recognized by the polyclonal antibody in most fractions, indicating a wide viral protein distribution along the glycerol-tartrate gradients. However, CMV proteins were mostly detected in fractions 3, 5–6 and 9–11 from the fibroblast gradient profile, while labeling was mostly restricted to fractions 1–5 and 11 in the epithelial profile. Gradient fraction 3 was the most immuno-labeled in both cell types, followed by fraction 5, which correlated with the Coomassiestaining. In addition, the most abundant protein band (Figure 5a,b) corresponded to the tegument protein pp65 that migrated to the expected size (63 kDa, indicated with a black arrowhead).

**Figure 6:**
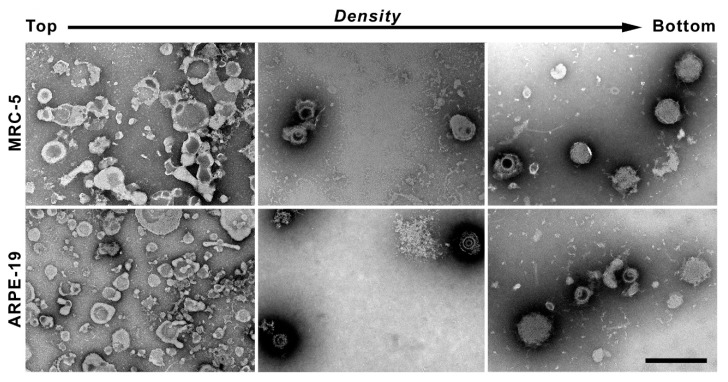
Electron microscopy of samples collected from glycerol-tartrate gradients. Representative images of each of the three parts of the gradients: top (**left panel**), middle (**central panel**) and bottom (**right panel**); indicating the main components isolated in each section in both MRC-5 and ARPE-19 cells. The top fraction was mainly integrated by cell membranes; the intermediate part was formed mostly by virions while DB mainly formed the bottom section.

**Figure 7:**
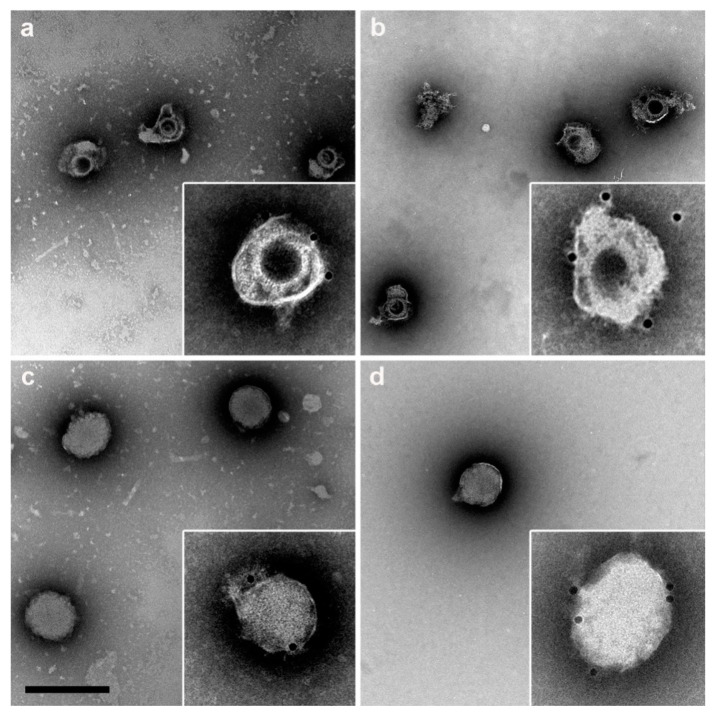
Electron microscopy of CMV assemblies. Transmission electron microscopy of negatively-stained virions (**a**,**b**) and DB (**c**,**d**) purified using glycerol-tartrate gradients from MRC-5- (**a**,**c**) and ARPE-19- (**b**,**d**) infected cells. The insets show immunoelectron microscopy images of the assemblies labeled with a polyclonal antibody anti-CMV. Bars: 500 nm.

Each of the 11 fractions were used for negative staining and analyzed by TEM in order to visualize the global distribution of the different viral (virions, non-infectious empty particles and DB) and non-viral assemblies (mostly membranes and debris). In general, the top fractions (from 1 to 4) mainly consisted of cell lipid membranes (Figure 6, left panels) and protein detection by Western blot may be recognizing viral proteins embedded in the membranes of the infected cells. The intermediate fractions (from 5 to 8) mostly contained a heterogeneous mixture of virions, with different integrity, with few DB particles and cell membrane debris (Figure 6 central panels ). The penetration of the staining agent revealed virions with a nucleocapsid structure surrounded by the tegument and the viral envelope, while DB appeared as dense spherical structures coated by a membrane with no nucleocapsid (Figure 7). The lower fractions (from 9 to 11) had the highest concentrations of DB particles in both MRC-5 and ARPE-19 infected cell gradients (Figure 6 central panels).

Figure S3 shows the size distribution of the purified DB along the size range, and representative images of the purified DB of the different sizes were also included. The size analysis of DB obtained from both MRC-5 and ARPE-19 cells showed significant differences (*p*-value = 4.31 × 10^−4^). While the size of ARPE-19-derived DB was smaller (223 ± 62 nm) and more homogeneous, DB obtained from MRC-5 infected cells were more heterogeneous (256 ± 90 nm), ranging from 100 to 600 nm. This correlated with the wider protein staining distribution of the MRC-5 DB gradient (fractions 9 to 11) relative to ARPE-19.

For further characterization, the bands containing virions and DB were collected from the gradients by lateral puncture and were used for immunodetection using the anti-CMV polyclonal antibody and analyzed using immunoelectron microscopy (Figure 7, insets). Negative control samples with no primary antibody were used to discard non-specific labeling of the secondary antibody. Both virions (Figure 7a,b, insets) and DB (Figure 7c,d, insets) isolated from MRC-5- and ARPE-19-infected cells were specifically recognized by the polyclonal CMV-specific antibody, indicating the viral nature of the isolated DB particles and confirming that DB are produced upon CMV infection from ARPE-19 epithelial infected cells.

## 4. Discussion

Although the in vivo function of the DB particles is unclear during CMV infection, these assemblies are only present in the cytoplasm co-localizing with viral particles. This may indicate that DB are produced in excess as an intermediate material during viral formation and may play a role in viral envelopment [45]. While capsid-tegument complexes are formed and released during the tegument formation in the nucleus, pp65 and other viral proteins are exported from the nucleus to the cytoplasm where they are assembled to form DB that are finally enveloped and released [45,46].

Several authors have highlighted the immunogenic potential of CMV DB due to their protein complexity and lack of infectivity [7,12,13,14], making them ideal vaccine candidates; however, no DB-based vaccine has been licensed [19]. Most of the previous work characterizing DB was performed in fibroblasts infected with different CMV strains [7,12,13,14,16,35]. It was also recently described the production of DB from CMV-infected amniotic fluid cells, pointing out that the number of viral particles and DB observed was very low, and that the infection of these cells was independent of the PC proteins [47]. Entry into epithelial, endothelial and myeloid cells mainly occurs through the interaction of the proteins of the PC and receptor in the cell membrane [30] and reduced CMV transmission has been linked to CMV-specific neutralizing antibodies against the pentamer complex present in immunoglobulin preparations [48]. Since the composition of the viral particles may differ depending of the type of cells infected [49], the use of viral strains with a functional PC will be ideal for the infection of epithelial cells and the production of DB. Further proteomic characterization of the DB will be necessary in order to validate this hypothesis. Obtaining a DB-vaccine-based progeny containing the PC in the membrane will be ideal, since it may elicit a more significant neutralizing antibody response [10,14]. A possible issue regarding a DB-based vaccine is the potential contamination with aggregated infectious viral particles that could migrate to the DB fraction. In order to avoid virion contamination in the final DB preparations, several additional safety strategies can be used such as sample irradiation with UV light prior to gradient centrifugation orvirus attenuation by deleting of UL25 gene from the genome, among others [10].

In addition, although the extent of the participation of NK cells in the vaccination response is unclear, new evidence suggests that NK cells may also play an important role post-vaccination contributing to either the induction and/or the effector phase of immunity [50,51,52,53,54,55]. Because of the protein complexity of the DB particles, a vaccine based on DB may more likely be able to induce different arms of the immune response, including NK cells. Recent studies have hypothesized a potential role of Th17 CD4 T lymphocytes (that secrete the IL-17 cytokine upon activation, among others [56,57,58]) during viral infection, promoting the secretion of pro-inflammatory cytokines and activating other immune cells [58]. The relevance of IL-17 has also been linked to several autoimmune and chronic disease such as Rheumatoid arthritis [59]. In this sense, Th17 cells are abundant in in the oral cavity, gastrointestinal tract, lungs, vagina, and skin, where they promote the integrity maintenance of the epithelial barrier [60]. Since epithelial cells are the main CMV target during natural infection, it may be interesting to study whether Th17 cells can be activated by a DB-based vaccine.

Ultrastructural analysis of both types of CMV-infected cell types shows a strong reorganization of cellular architecture in the nucleus and in particular areas of the cytoplasm. The examination of the perinuclear areas of the cytoplasm reveals the presence of a CI, a unique hallmark of CMV among herpesvirus [39]. These structures, known as the assembly complex, consist of redistributed organelles and components of the cellular secretory apparatus that support the final steps in CMV morphogenesis [34,43,61]. In this area, it is possible to distinguish viral capsids and DB in CMV-infected ARPE-19 cells that are also present in MRC-5 fibroblastsand it is in agreement with results from other studies using CMV-infected fibroblasts [16,35,43,62,63,64]. CMV capsids with or without a dense core (which will progress to virions and NIEPs) were present in the cytoplasm of the infected cells, as well as structures compatible with DB with or without envelope and were only present in the assembly complex. Non-enveloped DB were unequivocally distinguished from other particles of similar size; shape and density such as lysosomes or residual bodies because they are membranous organelles, and this is the first proof of DB assembly in the epithelial ARPE-19 cell line.

Although nuclear and cytoplasmic alterations in MRC-5- and ARPE-19-infected cells were similar, there were also qualitative differences such as the number of viral particles that were higher in the ARPE-19 assembly complex. Regarding nuclear morphology, large polykaryocytes, usually containing more than four nuclei, were only detected in CMV-infected ARPE-19 cells. Some of these nuclei show multiple electron-dense spherical bodies, compatible with nucleus condensation (pyknosis) in cells undergoing apoptosis. The differences observed between cell types cannot be attributed to the replication rate since ARPE-19 cells have a doubling time approximately of 24 h [65], while in our experience, the doubling time for MRC-5 cells is approximately 48h and maybe attributable to differences in the morphogenesis in both cell types. Additionally, although the formation of multinucleated cells has been reported in other fibroblast cell lines during infection [62], cell fusion was less common in MRC-5 infected cells, and polycaryocytes were observed rarely and never with more than 3 or 4 nuclei.

Most of the previous studies characterizing DB in fibroblasts used morphological criteria [13,14,16]. Here, we were able to purify and identified the CMV-derived particles released to the supernatant from infected fibroblast and those produced in ARPE-19 epithelial cells using immunoelectron microscopy. Gradient fraction distribution and biochemical and structural analysis suggested that the virions derived from epithelial infected cells have similar sedimentation pattern compared with those from fibroblasts [13,14,32,33]. However, epithelial cell-derived DB have higher density compared with fibroblast-derived DB, which may indicate differences in protein composition Although based on the negative staining, the morphology of DB particles was very similar in both cell types, ARPE-19-derived DB were more homogeneous in size (200–300 nm) compared with MRC-5-derived DB (100–600 nm). In agreement with previous results characterizing DB from CMV-infected fibroblasts [8,9], the tegument protein pp65 was also predominant in ARPE-19-derived DB, which were also coated by a phospholipid bilayer including the major viral envelope protein complexes [66].

Our results evidence the effective assembly and release of DB in ARPE-19 epithelial cells when infected with a derivative of the AD169 strain that expresses the PC, which may be a suitable alternative for developing a multi-protein vaccine against CMV. Further characterization is required to determine protein composition and the biological function of these viral particles during infection.

## Supplementary Materials

The following supporting information can be downloaded at: https://www.mdpi.com/article/10.3390/vaccines10081308/s1; Figure S1. Viral production kinetics of MRC-5 and ARPE-19 cells CMV-infected (MOI of 0.01) at 1, 5 and 10 days postinfection. The cells and medium were harvested at the indicated times and frozen at -80°C. The virus titer was de-termined in triplicate by qPCR and graphed as log10 mean value± SD. *Significant differences (p ≤ 0.05). Figure S2. Glycerol tartrate gradients of MRC-5 and ARPE-19 infected cells. The gradient separation of virions, DB, and noninfectious particles (NIEPs) are indicated. Representative images of the gradients after ultracentrifugation. Figure S3. Histograms representing size distribution of the purified DB from de gradient fractions 9-11 in MRC-5 and ARPE-19 cells. Bars represent the number of the DB with a similar diameter value within the same range of variation (50 µm). Insets show representative.
