# Supplementary material for: Identification and Characterization of Epithelial Cell-Derived Dense Bodies Produced upon Cytomegalovirus Infection
Source: Vaccines (Basel). 2022 Aug 12;10(8):1308. doi: 10.3390/vaccines10081308 (PMC9412340; doi:10.3390/vaccines10081308)
Supplement: Supplementary file 1 [file vaccines-10-01308-s001.zip › vaccines-1838055-supplementary.pdf]

vaccines

MDPI

Supplementary Materials

Identification and Characterization of Epithelial Cell-Derived Dense Bodies Produced upon Cytomegalovirus Infection

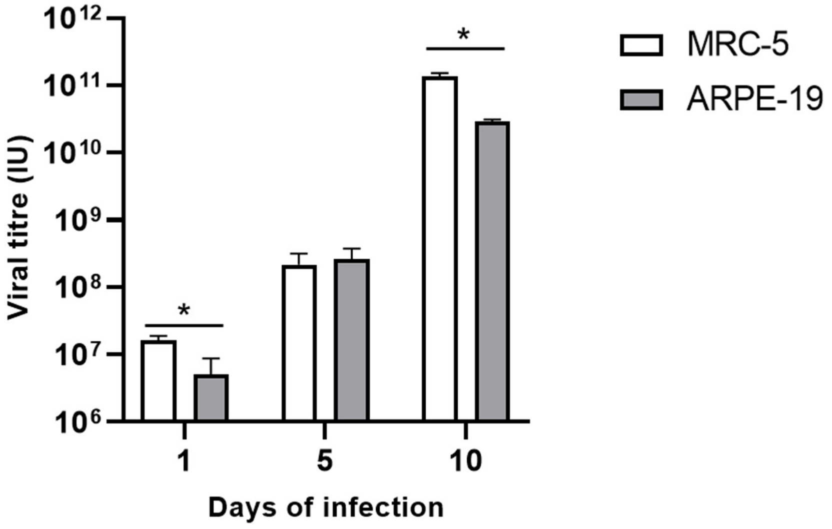

Figure S1. Viral production kinetics of MRC-5 and ARPE-19 cells CMV-infected (MOI of 0.01) at 1, 5 and 10 days postinfection. The cells and medium were harvested at the indicated times and frozen at -80°C. The virus titer was determined in triplicate by qPCR and graphed as log10 mean value± SD. *Significant differences ($p \leq 0.05$).

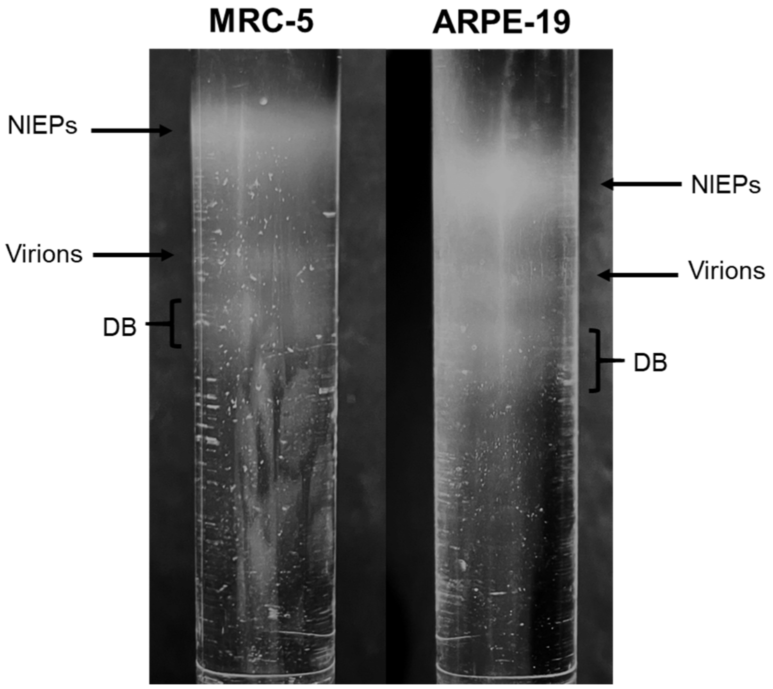

Figure S2. Glycerol tartrate gradients of MRC-5 and ARPE-19 infected cells. The gradient separation of virions, DB, and noninfectious particles (NIEPs) are indicated. Representative images of the gradients after ultracentrifugation.

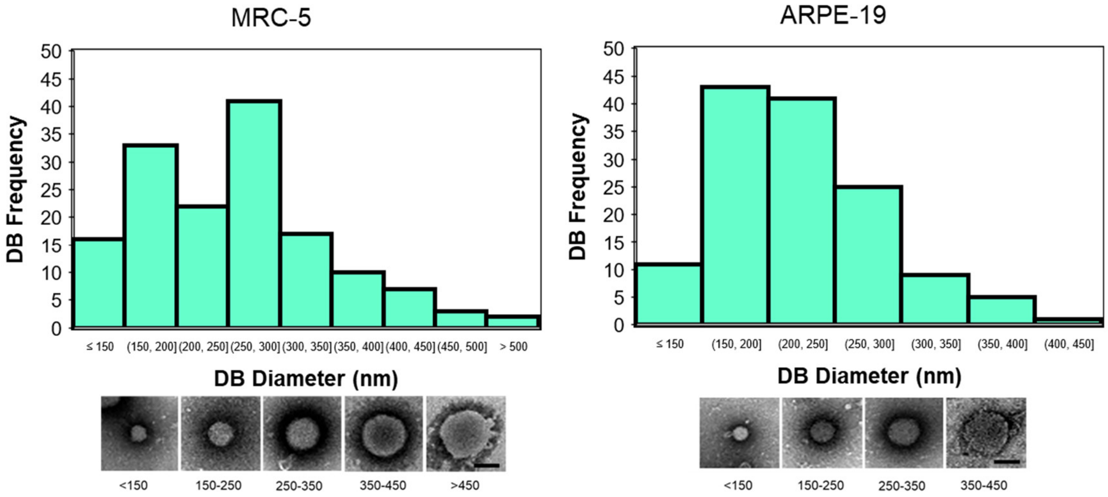

Figure S3. Histograms representing size distribution of the purified DB from de gradient fractions 9-11 in MRC-5 and ARPE-19 cells. Bars represent the number of the DB with a similar diameter value within the same range of variation (50 μm). Insets show representative.
